# Catalase Activity in the Brain Is Associated with Recovery from Brain Injury in a Piglet Model of Traumatic Brain Injury

**DOI:** 10.3390/brainsci15060608

**Published:** 2025-06-04

**Authors:** Stephanie T. Dubrof, Sarah L. Schantz, Taylor H. LePage, Sydney E. Sneed, Savannah R. Cheek, Holly A. Kinder, Kylee J. Duberstein, David A. DeWahl, Jerry O. Stern, Alexander B. Baguisi, Erin E. Kaiser, Franklin D. West, Hea Jin Park

**Affiliations:** 1 Department of Nutritional Sciences, University of Georgia, Athens, GA 30602, USA; 2 Regenerative Bioscience Center, University of Georgia, Athens, GA 30602, USA; sarah.schantz@uga.edu (S.L.S.);; 3 Biomedical and Health Sciences Institute, University of Georgia, Athens, GA 30602, USA; 4 Department of Animal and Dairy Science, University of Georgia, Athens, GA 30602, USA; 5 Ischemix, Inc., Grafton, MA 01519, USA

**Keywords:** swine model, antioxidants, oxidative stress, antioxidant enzymes, TBI, pediatric

## Abstract

**Background/Objectives**: Traumatic brain injury (TBI) is a global leading cause of disability and death, with millions of new cases added each year. Oxidative stress significantly exacerbates primary TBI, leading to increased levels of intracerebral cell death, tissue loss, and long-term functional deficits in surviving patients. Catalase and superoxide dismutase (SOD) mitigate oxidative stress and play a critical role in dampening injury severity. This study examines the neuroprotective effects of the novel antioxidant alpha lipoic acid-based therapeutic, CMX-2043, on antioxidant enzymes in a preclinical TBI model via various drug administration routes. **Methods**: Piglets (*n* = 28) underwent cortical controlled impact to induce moderate–severe TBI and were assigned to placebo (*n* = 10), subcutaneous CMX-2043 (SQ, 10 mg/kg; *n* = 9), or intravenous CMX-2043 (IV, 9 mg/kg; *n* = 9) treatment groups. Treatments began 1 h after TBI induction and continued for 5 days. MRI was performed throughout the study period to evaluate brain recovery. Blood was collected at 1, 7, and 42 days post-TBI, and liver and brain tissues were collected at 42 days post-TBI to measure catalase and SOD activity. **Results**: CMX-2043 IV-treated piglets showed 46.3% higher hepatic catalase activity than placebo (*p* = 0.0038), while the SQ group did not show significant changes in hepatic catalase activity compared to placebo. In the brain, SQ-treated piglets had significantly higher catalase activity than both IV (*p* = 0.0163) and placebo (*p* = 0.0003) groups (45.8340 ± 3.0855, 36.4822 ± 1.5558, 31.6524 ± 1.3129 nmol/min/mg protein for SQ, IV, and placebo, respectively), while IV-treated piglets did not show significant changes compared to placebo. IV-treated piglets did exhibit 39.3% higher brain SOD activity than placebo (*p* = 0.0148), while the SQ group did not show a significant change. CMX-2043 treatment did not alter plasma antioxidant enzyme activity during the study period. Importantly, within CMX-2043 treated TBI groups, piglets with significantly decreased lesion volumes, midline shift, and combined swelling and atrophy had better brain recovery, determined by MRI on day 1, 7, and 42 days post-injury TBI, exhibited higher brain catalase activity at 42 days post-injury TBI regardless of administration route, suggesting a link between improved recovery and sustained local catalase activity. **Conclusions**: This study highlights the impact of administration route on tissue-specific antioxidant responses, with IV administration enhancing liver catalase and brain SOD activity, while SQ administration primarily elevated brain catalase activity. In addition, this study shows an association between increased brain catalase activity and decreased TBI brain lesioning, midline shift, and combined swelling and atrophy, thus emphasizing the role of antioxidant defenses in neuroprotection post-injury.

## 1. Introduction

Traumatic brain injury (TBI) is a leading cause of disability and death worldwide, with millions of new cases added each year [1]. TBI tissue damage is a combined result of a complex injury and pathophysiology, including the primary and secondary injury pathways [2,3,4,5]. The primary injury is typically due to a mechanical insult such as a jolt or blow to the head leading to structural damage, hemorrhage, swelling, and atrophy in the brain [2,3]. This primary injury is quickly followed by the activation of a complex sequence of cellular and biochemical events that contribute to the secondary injury cascade [1,5,6,7]. Many of these processes are an attempt to restore homeostasis, but it often becomes dysregulated, resulting in oxidative stress, excitotoxicity, and inflammation that exacerbate neuropathology [1,5,6]. Specifically, oxidative stress occurs as a result of a loss of equilibrium between free radical formation and the antioxidant system, leading to a buildup of free radicals such as superoxide anion (O_2_^•−^), hydroxyl radicals (OH^•^), and hydrogen peroxide (H_2_O_2_) [6,8]. Moreover, the brain is more susceptible to oxidative damage due to a high amount of peroxidizable fatty acid content [6,9]. Antioxidants have gained increasing attention as a therapeutic approach for mitigating secondary brain injury due to their ability to neutralize ROS and preserve cellular integrity [10,11,12]. Following TBI, the rapid generation of ROS overwhelms the brain’s intrinsic antioxidant defenses, contributing to lipid peroxidation, mitochondrial dysfunction, and neuronal apoptosis [1,2,5]. A range of antioxidant compounds, including vitamin E, vitamin C, and alpha-lipoic acid (ALA), have been evaluated in both preclinical and clinical models of TBI with varying degrees of success [11,12,13,14]. While clinical trials have been limited and heterogeneous in outcomes, these studies underscore the therapeutic potential of targeting oxidative stress.

In response to injury, the brain upregulates genes that encode antioxidant enzyme systems, such as catalase and superoxide dismutase (SOD), which help terminate free radical reactions before there is widespread oxidative damage [15,16]. Catalase and SOD are key components of the brain’s physiological antioxidant defense system, playing essential roles in neutralizing reactive oxygen species (ROS) and mitigating oxidative damage [10]. These enzymes regulate redox balance and safeguard cells from oxidative injury, which is especially vital in the brain due to its high metabolic activity and lipid-rich composition [10,17,18]. In the aftermath of TBI, the production of ROS can overwhelm these endogenous antioxidant defenses [6,19]. As a result, the levels of catalase and SOD may serve as biomarkers of oxidative burden and have been associated with injury severity and prognosis in both experimental and clinical studies [20,21]. Understanding the regulation of these antioxidant enzymes in response to injury and therapeutic interventions is essential for developing neuroprotective strategies.

CMX-2043 is a novel ALA-based therapeutic compound that has neuroprotective, metabolic, and antioxidative properties that may limit TBI-induced tissue damage and long-term functional deficits [22]. ALA is a metabolic antioxidant proven to be a potent protector of neuronal cells from oxidative stress [11,22,23,24]. Specifically, studies have shown that ALA can enhance the activity of catalase and SOD [25]. This has been demonstrated in various models, including in vivo rodent studies of oxidative injury [26,27,28], in vitro cell culture systems [29], and clinical studies in patients with non-neurological conditions such as diabetes and chronic kidney disease, where systemic antioxidant effects have been observed [24,25]. However, CMX-2043 has demonstrated superior antioxidant potential than ALA in preclinical studies, showing enhanced efficacy in protecting cardiac cells from ischemia–reperfusion injury [22]. In this context, the antioxidant CMX-2043 is a promising candidate for mitigating TBI-associated oxidative damage.

Route of administration can affect drug bioavailability, distribution, and metabolism, ultimately influencing its capacity to reach target tissues [30,31]. Intravenous delivery ensures rapid systemic distribution, potentially leading to higher concentrations in circulation [32], whereas subcutaneous administration may result in slower absorption and prolonged release [33,34]. Examining how these differences may influence antioxidant enzyme activity and recovery outcomes is essential for optimizing treatment strategies for TBI [31]. Therefore, this study examines both subcutaneous and intravenous administration of CMX-2043 to compare therapeutic efficacy.

The piglet model is widely recognized for its translational value in TBI research due to its gyrencephalic brain structure, white-to-gray matter ratio, and developmental similarity to the pediatric human brain [35,36]. These features make it particularly suitable for evaluating both the pathophysiological response to injury and the effects of therapeutic interventions using clinically relevant imaging modalities such as MRI [36,37,38].

While previous research has shown that CMX-2043 enhances antioxidant capacity in vitro [22], its effects on enzymatic antioxidant activity within the brain remain unexplored. This study examines the effects of CMX-2043 administered both subcutaneously and intravenously on antioxidant enzyme activity in a preclinical pig TBI model. It also explores its potential role in neuroprotection by assessing the relationship between oxidative stress markers and MRI-based metrics of injury and recovery.

## 2. Materials and Methods

### 2.1. Animal Handling and Study Design

At six weeks of age, Yorkshire crossbred piglets (approximately 12–18 kg; *n* = 28) were randomly assigned to experimental groups: saline (placebo, 40 mg/kg; *n* = 10), subcutaneous administration of CMX-2043 (SQ, 10 mg/kg; *n* = 9), or intravenous administration of CMX-2043 (IV, 9 mg/kg; *n* = 9). Piglets were allowed ad libitum access to a corn-based diet formulated to meet their metabolic and nutritional requirements, in accordance with the National Research Council’s recommendations for swine nutrition [39]. The CMX-2043 dosing regimens were based on prior rodent studies demonstrating efficacy and safety in models of oxidative stress [22,27]. Doses were adjusted for body size and frequency was optimized to maintain antioxidant exposure during the acute TBI phase. While piglet-specific pharmacokinetics have not been formally published, the selected regimen aligns with subcutaneous absorption kinetics reported in other large mammals [40]. SQ piglets were given CMX-2043 beginning 1 h post-TBI and continued thereafter every 8 h for a total period of 5 days. SQ injections were administered by trained staff using needles about 25–75 mm below and behind the base of the pig’s ear, where the subcutaneous fat is accessible. IV piglets were given CMX-2043 beginning 1 h post-TBI and continued every 12 h for a total period of 5 days. CMX-2043 was administered via intermittent IV infusion over a 10 min period. Pigs were briefly anesthetized with isoflurane (VetOne, Boise, ID, USA) in oxygen for the 10 min period of CMX-2043 administration. Daily intravenous access was re-established as needed using either a peripheral catheter with an extension set or a butterfly catheter, depending on auricular vein condition. All procedures were performed by trained veterinary staff and did not require additional surgical preparation compared to the SQ group. The inclusion of both subcutaneous and intravenous administration allowed for the evaluation of potential differences in drug efficacy, which may influence treatment outcomes. Furthermore, the rationale for comparing SQ and IV routes was based on prior rodent studies using SQ CMX-2043 and the need to assess whether pharmacodynamic differences or tissue-specific enzyme responses emerged in a large-animal model with different absorption characteristics.

Piglets underwent MRI analysis at 1, 7, and 42 days post-TBI, using methods as previously described [36,41]. Briefly, multiplanar MRI sequences, including T2 Weighted (T2W), were acquired to assess lesion volume, hemispheric swelling, and atrophy [36,41]. Using T2W sequences, trained and blinded analysts manually identified regions of interest (ROIs), and OsiriX software (Version 12.5.2) calculated ipsilateral and contralateral hemisphere volumes (cm^3^), lesion volumes (cm^3^), and midline shift (MLS) by measuring the deviation from the ideal midline (mm) [36,41]. Swelling/atrophy was calculated as the difference between the ipsilateral and contralateral hemisphere volumes. Positive values indicated hemispheric swelling (when the ipsilateral hemisphere exceeded the contralateral in volume, typically at acute time points), whereas negative values reflected atrophy (when the ipsilateral hemisphere volume was reduced relative to the contralateral side, typically at later stages). Both were calculated using the same approach and are presented as a unified measure of structural change over time. This study was conducted in accordance with the University of Georgia Institutional Animal Care and Use Committee (IACUC) guidelines (Animal Use Protocol: A2022 08-005-Y1-A0).

#### 2.1.1. Controlled Cortical Impact of Traumatic Brain Injury

The TBI induction procedure was carried out using previously established methods [35,36,38,41,42], including controlled cortical impact to induce a moderate-severe TBI. Briefly, a 4 cm left-sided incision was made to expose the skull. A 20 mm craniectomy was performed over the motor cortex, and TBI was induced using a controlled cortical impact device with a 15 mm blunt impactor tip (4 m/s velocity, 9 mm depression depth, 400 ms dwell time).

#### 2.1.2. Plasma, Liver, and Brain Tissue Collection

**Figure 1:**
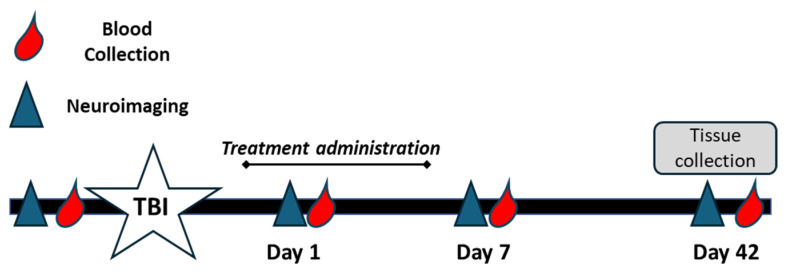
Experimental timeline for CMX-2043 treatment and sample collection. Piglets were subjected to controlled cortical impact to induce TBI (white star) and subsequently treated with CMX-2043 via intravenous (IV) or subcutaneous (SQ) administration for 5 days post-injury. Blood samples were collected at baseline (pre-TBI), and on days 1, 7, and 42 post-TBI (red droplets) to assess plasma antioxidant activity. MRI scans were conducted at the same time points to evaluate brain injury and recovery (blue triangles). Tissue samples (brain and liver) were collected on day 42 post-TBI for enzymatic analysis.

Blood samples were collected from all piglets via jugular vein access into EDTA tubes to isolate plasma. Blood was collected at baseline (pre-TBI), immediately prior to treatment administration on day 1 post-TBI, and again at 7 and 42 days post-TBI (Figure 1). Plasma samples were flash frozen in liquid nitrogen and stored in −80 °C until analysis.

At 42 days post-TBI, all piglets were humanely euthanized, and liver and brain tissues were collected. Liver and brain tissue samples were immediately flash frozen in liquid nitrogen and stored at −80 °C until analysis. Euthanasia was performed in accordance with IACUC standards.

### 2.2. Measurement of Catalase and Superoxide Dismutase Activity in Plasma and Tissue

Catalase and SOD activity in plasma, liver, and brain tissue were assessed to evaluate antioxidant defense in piglets. Tissue samples from the liver and brain were homogenized in potassium phosphate buffer for the catalase assay and in HEPES buffer for the SOD assay. Catalase and SOD activity were measured using the catalase assay kit (Cayman, Ann Arbor, MI, USA) and SOD assay kit (Cayman, Ann Arbor, MI, USA), following the manufacturer’s instructions. Protein content of the homogenates was quantified using the Pierce BCA protein assay (Thermo Fisher Scientific, Waltham, MA, USA), and catalase and SOD activity were normalized to protein content.

### 2.3. Statistical Analysis

Treatment effects were analyzed using one-way ANOVA. Time-dependent changes in plasma catalase and SOD activity across the time points were analyzed using repeated measures ANOVA. Data are expressed as the mean ± S.E.M. Pearson correlations were performed to determine relationships between brain catalase and SOD activity with MRI measurements that were obtained following TBI, including midline shift (mm), lesion volume (cm^3^), and swelling/atrophy (cm^3^). To further investigate the relationship between antioxidant activity and brain preservation and recovery, MRI measurements of CMX-2043-treated piglets were stratified into top and bottom half groupings and analyzed in relation to catalase activity, allowing for a focused assessment of antioxidant levels and their potential link to MRI outcomes. This analysis was limited to treated animals to isolate the effects of CMX-2043, as the drug had a significant impact on MRI outcomes. Including placebo animals could have introduced confounding from untreated injury, thereby obscuring treatment-related associations. An unpaired *t*-test was used to assess for differences in catalase activity in the brain in treated piglets based on these MRI-defined recovery groups. All analyses were performed using GraphPad Prism (Version 10.1.0, GraphPad Software, Inc.; San Diego, CA, USA).

## 3. Results

### 3.1. Antioxidant Enzyme Activity in Plasma Changed over Time Following TBI

**Figure 2:**
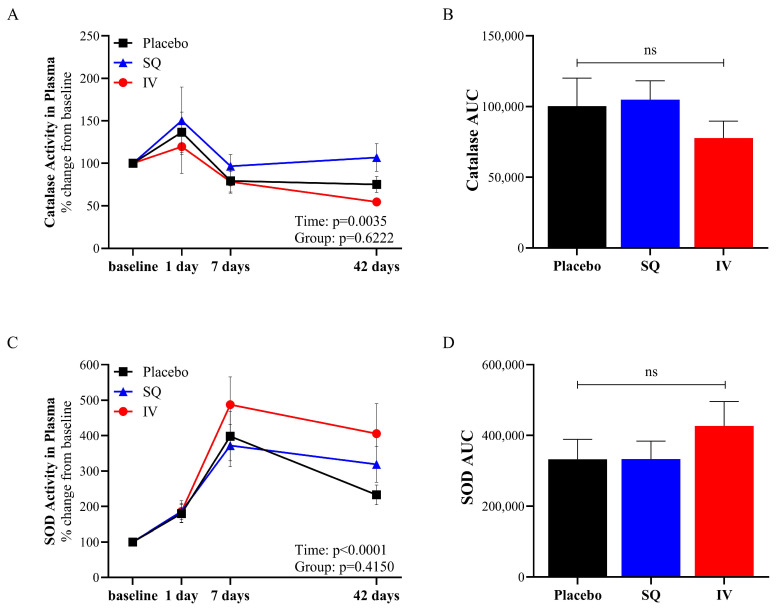
Antioxidant enzymatic activity in plasma from placebo (*n* = 10), SQ (*n* = 9), and IV (*n* = 9) piglets following TBI induction. Catalase activity in plasma (**A**), AUC calculated from catalase activity in plasma from baseline to 42 days post-TBI (**B**), SOD activity in plasma (**C**), and AUC calculated from SOD activity in plasma from baseline to 42 days post-TBI (**D**). AUC: area under the curve; SOD: superoxide dismutase; SQ: subcutaneous treatment group; IV: intravenous treatment group.

Catalase and SOD activity was measured in plasma collected from piglets at baseline (pre-TBI) and on days 1, 7, and 42 following TBI (Figure 2). Catalase exhibited temporal changes following TBI, with an initial increase followed by a decline over time in all three groups (Figure 2A). The area under the curve (AUC) analysis for catalase activity (Figure 2B) revealed no significant differences between the treatment groups, suggesting that CMX-2043 administration did not significantly alter overall catalase activity in plasma. SOD activity (Figure 2C) also showed an early (1 and 7 days) increase post-TBI and a decrease at 42 days but still remained elevated relative to baseline. However, the AUC analysis for SOD activity (Figure 2D) again showed no significant differences between treatment groups, further reinforcing that the treatment did not significantly alter overall SOD activity in plasma.

Despite the lack of significant treatment effect, there was a significant effect of time on antioxidant enzymatic activity in plasma following TBI. This indicates that while treatment did not directly influence plasma antioxidant activity, the temporal changes observed may reflect the natural physiological response associated with limiting TBI and promoting recovery.

### 3.2. Administration Route of CMX-2043 Differentially Altered Antioxidant Status in Liver and Brain Following TBI

**Figure 3:**
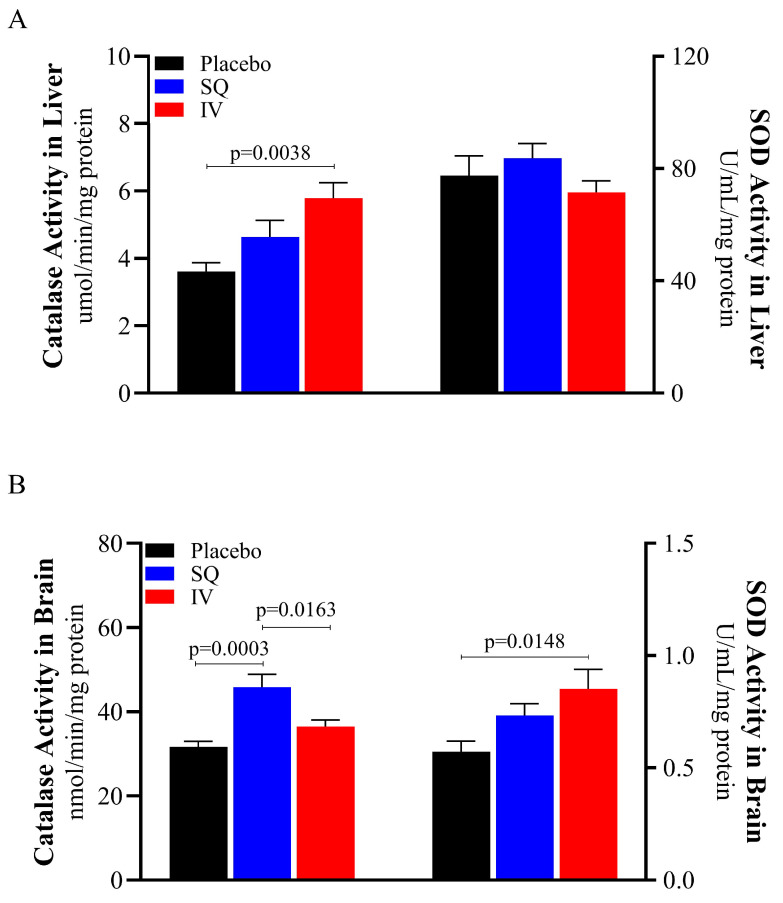
Antioxidant enzymatic activity in liver and brain from piglets at 42 days post-TBI. Catalase and SOD activity in liver (**A**), catalase and SOD activity in brain (**B**). SOD: superoxide dismutase; SQ: subcutaneous treatment group; IV: intravenous treatment group.

Catalase and SOD activities were assessed in both the liver and brain to evaluate tissue-specific antioxidant responses following TBI (Figure 3). CMX-2043 increased hepatic catalase activity, as well as catalase and SOD activity in the brain. Interestingly, the administration route of CMX-2043 influenced catalase and SOD activity differently in brain and liver tissues. Specifically, IV piglets exhibited 46.3% higher catalase activity in the liver than placebo (5.7900 ± 0.4518 μmol/min/mg protein and 3.6126 ± 0.2608 μmol/min/mg protein for IV and placebo, respectively; *p* = 0.0038), while the SQ group was not statistically different (4.6410 ± 0.4894 μmol/min/mg protein; *p* > 0.05) (Figure 3A). SOD activity in the liver did not differ significantly between SQ, IV, and placebo treatment groups (83.6760 ± 5.2844, 71.5300 ± 4.1467, 77.4373 ± 7.0645 U/mL/mg protein, respectively; *p* > 0.05).

The administration route of CMX-2043 also differentially affected catalase and SOD activity in the brain (Figure 3B). SQ-treated piglets had significantly higher brain catalase activity than both IV (*p* = 0.0163) and placebo (*p* = 0.0003) groups (45.8340 ± 3.0855, 36.4822 ± 1.5558, 31.6524 ± 1.3129 nmol/min/mg protein for SQ, IV, and placebo, respectively). IV piglets’ brain catalase activity was not different from placebo (*p* > 0.05). However, brain SOD activity in IV piglets was 39.3% higher than placebo piglets (0.8522 ± 0.0875 and 0.5722 ± 0.0475 U/mL/mg protein for IV and placebo, respectively; *p* = 0.0148), while the SQ group did not show a significant difference relative to placebo (0.7330 ± 0.0521 U/mL/mg protein; *p* > 0.05). These findings demonstrate that CMX-2043 enhances antioxidant enzyme activity in a tissue and delivery dependent manner, as IV administration increased catalase activity in the liver and SOD activity in the brain, while SQ administration primarily elevated brain catalase activity.

Additionally, catalase and SOD activity in the brain was notably lower than activity levels in the liver (Figure 3), thus supporting previous literature that characterized antioxidant activities in the mouse brain and liver, where brain antioxidant activity was substantially lower than liver antioxidant activity [43]. As the primary site of systemic antioxidant metabolism, the liver plays a well-established role in regulating oxidative balance and detoxification, making it a critical reference tissue when evaluating antioxidant interventions following TBI. Moreover, the brain’s heightened vulnerability to oxidative damage is partly due to its relatively weak antioxidant defense systems, which is consistent with the lower antioxidant activity levels observed [1,20].

### 3.3. Catalase Activity in the Brain Is Associated with Decreased Brain Tissue Damage Following TBI

CMX-2043 treatment significantly reduced MRI-derived markers of brain injury across multiple time points when compared to placebo piglets. (*p* < 0.001). For midline shift, SQ-treated animals showed a 57% reduction at day 1, 41% at day 7, and 50% at day 42, while IV-treated animals showed a 50% reduction at day 1, 30% at day 7, and 51% at day 42 (*p* < 0.001). Regarding lesion volume, SQ treatment resulted in a 44% reduction at day 1, 67% at day 7, and 71% at day 42, whereas IV treatment produced a 48% reduction at day 1, 67% at day 7, and 67% at day 42 compared to placebo piglets (*p* < 0.0001). For swelling/atrophy, which reflects hemispheric volume changes, SQ-treated animals showed a 65% reduction in swelling at day 1 and a 43% reduction in atrophy at day 42, while IV-treated animals showed a 70% reduction in swelling at day 1 and a 50% reduction in atrophy at day 42 compared to the placebo group (*p* < 0.001). These results highlight the consistent and substantial effect of CMX-2043, via both subcutaneous and intravenous routes, on reducing structural damage and preserving brain tissue following TBI.

**Figure 4:**
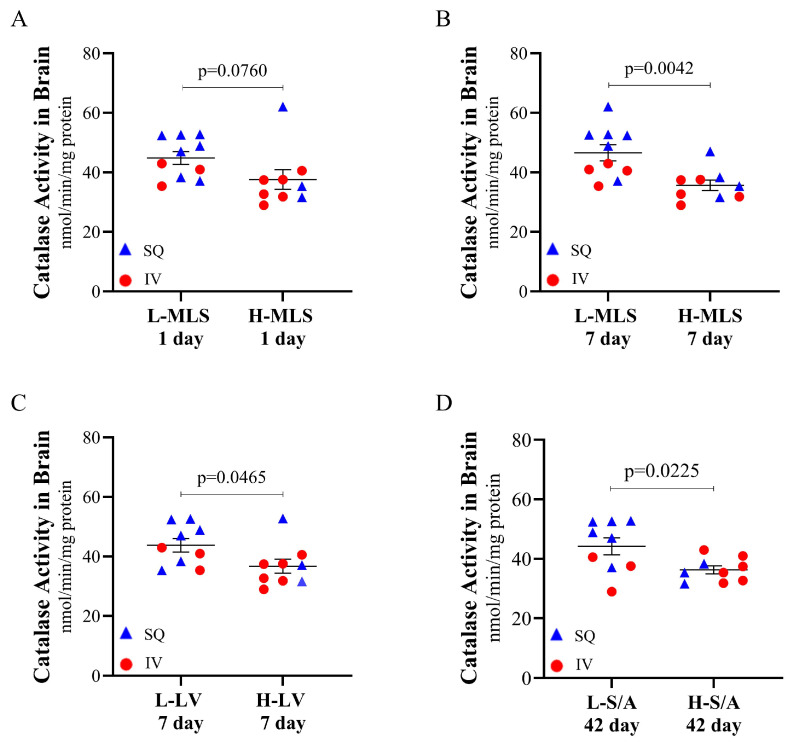
Catalase activity at 42 days post-TBI in the brain of CMX-2043-treated piglets stratified by MRI-defined injury severity groups. Catalase activity is stratified by low and high 1 day midline shift (**A**), low and high 7 day midline shift (**B**), low and high 7 day lesion volume (**C**), and low and high 42 day swelling/atrophy (**D**) in piglets receiving CMX-2043 treatment (*n* = 19). L-: low group; H-: high group; MLS: midline shift; LV: lesion volume; S/A: swelling/atrophy; SQ: subcutaneous treatment group; IV: intravenous treatment group.

To evaluate the relationship between antioxidant status and tissue damage within the brain post-TBI, catalase and SOD activity values were assessed against MRI measurements in piglets to identify correlations (Table 1). It was observed that catalase activity in the brain was negatively correlated with multiple MRI measurements, including 1 day midline shift (r = −0.4557, *p* = 0.0148), 7 day midline shift (r = −0.6241, *p* = 0.0004), and 42 day midline shift (r = −0.5448, *p* = 0.0033). There was a trending negative correlation between catalase activity in the brain with 1 day (r = −0.3433, *p* = 0.0860) and 7 day (r = −0.3419, *p* = 0.0749) lesion volume, as well as a significant negative correlation with 42 day lesion volume (r = −0.5770, *p* = 0.0016). A significant negative correlation between catalase activity and 1 day swelling/atrophy was also observed (r = −0.4084, *p* = 0.0383). To further investigate this, an analysis of only piglets treated with CMX-2043 (*n* = 19) was conducted to substantiate if there was a relationship between catalase activity and MRI markers of TBI. Piglets were stratified into two groups based on midline shift, lesion volume, and swelling/atrophy severity using MRI measures, creating “low” and “high” groups for each parameter (Figure 4). The catalase activity was then compared between low and high groups within a given parameter allowing for a more focused assessment of catalase activity across varying degrees of injury. The results indicated that among CMX-2043-treated piglets, those with improved MRI-assessed markers of brain injury also exhibited higher catalase activity in the brain, independent of administration route. Specifically, catalase activity in the brain trended higher in the low 1 day midline shift group (44.8512 ± 2.1394 nmol/min/mg protein) compared to the high group (37.5749 ± 3.2969 nmol/min/mg protein; *p* = 0.0760) (Figure 4A). Catalase activity in the brain was significantly higher in the low 7 day midline shift group (46.5803 ± 2.6927 nmol/min/mg protein) versus the high group (35.6537 ± 1.7765 nmol/min/mg protein; *p* = 0.0042) (Figure 4B). Similarly, catalase activity in the brain was higher in the low 7 day lesion volume group (43.7768 ± 2.2649 nmol/min/mg protein) relative to the high group (36.7293 ± 2.3542 nmol/min/mg protein; *p* = 0.0465) (Figure 4C), as well as in the low 42 day swelling/atrophy group (44.2151 ± 2.8407 nmol/min/mg protein) compared to the high group (36.2910 ± 1.3349 nmol/min/mg protein; *p* = 0.0225) (Figure 4D). These findings demonstrate that within CMX-2043-treated piglets, higher catalase activity in the brain suggested reduced TBI-induced neural injury based on MRI biomarkers. Importantly, this relationship was independent of the route of administration (SQ or IV), suggesting that catalase brain activity may serve as a potential marker of recovery following treatment. Furthermore, catalase activity in the brain at 42 days post-TBI, which is also long after the final treatment, remained a predictor of recovery across the multiple MRI time points. These results also emphasize the variability in antioxidant responses and the need for further investigation to establish a causal link between antioxidant enzyme activity and brain recovery post-TBI.

## 4. Discussion

CMX-2043 treatment significantly increased hepatic catalase activity, as well as brain catalase and SOD activity in a piglet TBI model. Additionally, the effects of CMX-2043 on antioxidant enzyme activity in tissue varied depending on SQ or IV administration, influencing catalase and SOD activity differently in the liver and brain. Notably, our findings showed that higher catalase activity in the brain at 42 days post-TBI was related to improved MRI-based biomarkers of brain injury in CMX-2043-treated piglets, regardless of administration route. CMX-2043 treatment did not affect plasma antioxidant enzyme activities, although TBI itself altered these systemic markers throughout the study period. This is the first study to show that CMX-2043 treatment leads to increased levels of catalase and SOD brain activity in a post-TBI large animal model and that increased levels of brain catalase is associated with decreased brain injury. These findings suggest a potential relationship between CMX-2043-induced antioxidant activity and structural markers of brain recovery, warranting further investigation in larger, controlled studies.

**Figure 5:**
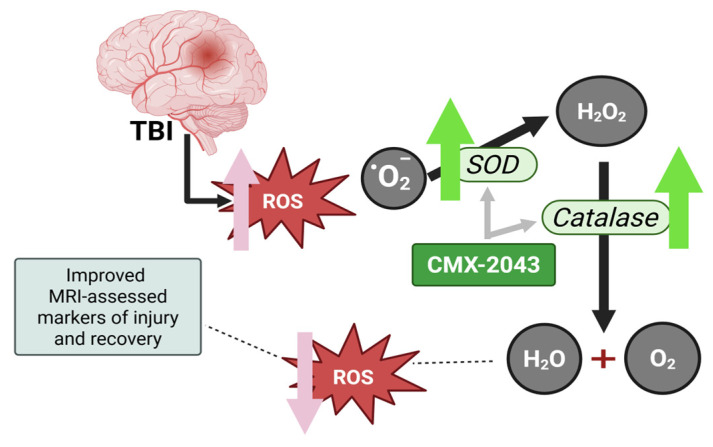
Schematic representation of the antioxidant response after TBI, and the proposed mechanism of action of CMX-2043. TBI leads to the generation of reactive oxygen species, including superoxide anions (O_2_^•−^), which contribute to oxidative damage. SOD catalyzes the conversion of superoxide into hydrogen peroxide (H_2_O_2_), which is subsequently detoxified into water (H_2_O) and oxygen (O_2_) by catalase and glutathione peroxidase. CMX-2043 enhances the activity of SOD and catalase and has superior radical-scavenging capacity thereby supporting oxidative stress regulation and neuroprotection following TBI. ROS: reactive oxygen species; SOD: superoxide dismutase.

Catalase is a key endogenous antioxidant enzyme that helps remove ROS and mitigate oxidative damage following TBI [10]. Physiologically, superoxide anion is rapidly and efficiently converted into H_2_O_2_ + O_2_ by the enzyme SOD, and H_2_O_2_ is then detoxified into O_2_ + H_2_O mainly by glutathione peroxidase and, partly, by catalase [17,18] (Figure 5). This enzymatic process involving catalase and SOD is essential for mitigating the detrimental effects of oxidative damage [18] and promoting recovery [19] after TBI. Maintaining the proper function and activity levels of these antioxidant enzymes is therefore crucial for facilitating the brain’s recovery [1,3,6]. This study demonstrates that higher brain catalase activity correlates with improved MRI measures, suggesting a potential link between antioxidant enzyme activity and TBI recovery. While this association was independent of administration route, SQ administration may offer logistical advantages in acute settings, as it can be administered more rapidly and without the need for trained personnel, unlike IV treatment which typically requires trained medical staff [44]. Earlier intervention is vital given the rapid onset of secondary injury following TBI. Although direct measurement of brain catalase requires invasive, post-mortem tissue collection, these findings highlight the critical role of antioxidant defenses in neuroprotection and the importance of oxidative stress regulation in recovery [1,3,45]. To address this limitation, non-invasive biomarkers that reflect similar oxidative stress responses should be explored. Emerging techniques such as magnetic resonance spectroscopy (MRS) offer a promising approach for assessing oxidative stress-related metabolites in the brain, enabling evaluation at earlier stages of recovery [46,47].

Antioxidants, often supplemented through diet [14], can diminish the potency of various oxidants [10,12,17]. Although endogenous antioxidants can mitigate free radical damage, elevated free radicals can overwhelm the body’s natural defenses, thus supplementing the brain’s antioxidant capacity may help prevent and alleviate oxidative damage during injury [12,14]. Studies have shown that alpha-lipoic acid, the analog of CMX-2043, can increase the activity of the antioxidant enzymes catalase and SOD in various models of diseases linked to oxidative stress, such as kidney and heart conditions [24,25,28]. In the liver of experimental hyperoxaluric rats, administration of ALA was found to enhance catalase activity and decrease peroxidative levels [26]. Similarly, ALA supplementation significantly increased serum SOD activity in patients undergoing hemodialysis [25], and markedly restored SOD activity in both the serum and renal cortex of diabetic rats [28]. CMX-2043 is composed of ALA covalently linked to a dipeptide adduct molecule [22]. In an investigation evaluating the antioxidant capacity of CMX-2043, ALA, and the dipeptide adduct individually, CMX-2043 demonstrated superior efficacy in scavenging peroxyl radicals, as measured by the oxygen radical absorbance capacity assay [22]. Notably, the dipeptide adduct had minimal antioxidant activity [22]. These findings suggest that the structural combination of ALA with the dipeptide is essential for enhancing the overall antioxidant potential of CMX-2043 [22]. The current study demonstrated that CMX-2043 treatment increased hepatic catalase activity and increased catalase and SOD activity in the brain, reinforcing the potential of the novel antioxidant molecule of CMX-2043 to restore antioxidant enzyme function under oxidative stress conditions.

Among CMX-2043-treated animals, higher catalase activity was associated with more favorable MRI markers of injury and recovery. Prior research has shown that enhanced activity of endogenous antioxidants, such as SOD and glutathione peroxidase, correlate with reduced oxidative damage and improved neuroprotection in both preclinical and clinical settings [10,48,49]. Despite this association, there remains no established approach for the use of antioxidants in the post-injury period to alleviate the effects of TBI [14]. Many studies have assessed the efficacy of other antioxidants to reduce TBI-associated oxidative damage in animal models and in a limited number of clinical trials as reviewed by Di Pietro et al. [14]. For example, vitamin E is a powerful antioxidant, as it can aggressively scavenge reactive oxygen species [13]. In a rodent model of TBI, Wu et al. found that vitamin E supplementation following TBI significantly normalized levels of oxidative markers, including SOD [13]. Administration of quercetin, another antioxidant, in a rodent TBI-model reduced cognitive deficit and increased catalase and SOD antioxidant activity in the hippocampus [50]. In a rat model of TBI assessing the effects of the antioxidant hydroxysafflor yellow A, it was observed that hydroxysafflor yellow A reduced markers of oxidative stress as observed through increased activity of both catalase and SOD in the brain [51]. These results fall in line with what was observed in the current study, as we also observed changes in enzymatic activity in the liver and brain in response to antioxidant administration. While the findings from this study contribute to the growing body of evidence, further work is needed to establish a rigorous protocol for antioxidant administration in the acute phase post-TBI.

The route of administration of CMX-2043 had distinct effects on antioxidant enzyme activity in the liver and brain. When administered SQ, a drug may be more readily absorbed and metabolized in the subcutaneous adipose tissue before reaching the liver and brain [30,34,52]. This could result in lower levels of the active drug reaching the intended tissue compared to IV administration, which would allow the drug to more directly access these organs due to increased bioavailability [30,53]. Studies have shown that differences in pharmacokinetics can impact drug bioavailability and distribution, ultimately influencing its therapeutic efficacy in different organs [54,55]. For instance, in a rodent study, IV administration of ALA resulted in higher peak plasma levels compared to oral intake, though both routes shared comparable elimination half-lives [56]. Another rodent study revealed that IV administration of CMX-2043 showed rapid clearance of the drug, exhibiting a half-life in rats of about 10 min [27]. Conversely, subcutaneous (SQ) administration involves ALA absorption through the interstitial tissues, leading to a slower onset and prolonged presence in circulation. In California sea lions, a single SQ dose of 10 or 20 mg/kg ALA peaked within 20 to 30 min, with a half-life of 40 and 32 min for 10 and 20 mg/kg doses, respectively [40]. Our results specifically indicated IV administration enhanced catalase activity in the liver and SOD activity in the brain, while SQ administration increased catalase activity in the brain, suggesting the administration route can differentially impact the ability of antioxidant therapies to modulate enzymatic activity in key tissues.

Though CMX-2043 treatment did not alter catalase or SOD activity in the plasma, a significant overall time difference was observed in the antioxidant activity of the plasma following TBI. In a cohort of patients with subarachnoid hemorrhage, SOD concentrations in plasma increased from day 1 to day 7 [57]. This falls in line with the observations in our study, where there is an apparent increase in SOD activity on day 7 before it begins to return to normal levels. Among ROS, the superoxide anion is the first to be produced after TBI by cerebral cells via multiple mechanisms, but mainly through the malfunctioning of the mitochondrial electronic transport chain [58]. This may explain why plasma SOD activity on day 1 was 83% higher than baseline, while plasma catalase activity increased by only 35% from baseline on the same day following TBI.

A limitation of this study is the absence of a non-TBI (sham) control group for liver and brain antioxidant enzyme measurements, which restricts interpretation of post-injury changes relative to physiological baseline. Consequently, conclusions drawn regarding tissue-specific catalase and SOD activity are based on relative comparisons among TBI-affected groups rather than absolute deviations from uninjured physiology. Additionally, while six-week-old piglets were selected for their developmental resemblance to the pediatric human brain in terms of neuroanatomy, it is important to note that age-specific pharmacokinetic data for CMX-2043 in piglets is currently lacking. As such, the dose regimen used here was extrapolated from prior rodent studies and scaled for a large-animal model.

In conclusion, this study demonstrates a dynamic relationship between brain catalase activity and improved MRI markers of injury following TBI. Moreover, CMX-2043 treatment increased brain catalase levels, which was associated with reduced lesion volume, midline shift, and swelling/atrophy. These findings reinforce the critical role of antioxidant defenses in neuroprotection and post-injury repair. Importantly, CMX-2043 treatment enhanced antioxidant enzyme activity in the liver and brain, with its effects varying by administration route, thus highlighting the importance of delivery method in optimizing therapeutic efficacy for TBI recovery. Future research should focus on elucidating the causal pathways linking antioxidant responses to structural and functional recovery, exploring the integration of enzymatic biomarkers with imaging modalities, and leveraging advanced analytics to enhance prognostic and therapeutic strategies. By addressing these gaps, we can advance our understanding of TBI pathology and develop more targeted interventions to improve outcomes.

## Figures and Tables

**Table 1:** Correlations of MRI measurements with catalase activity in the brain 42 days post-TBI. Pearson’s correlations between MRI measurements of midline shift, lesion volume, and swelling/atrophy at 1, 7, and 42 days post-TBI and catalase activity in the brain of all piglets (*n* = 28). r = Pearson’s correlation coefficient.

MRI Measurement	r	*p*-Value
1 Day Midline Shift	−0.4557	0.0148
7 Day Midline Shift	−0.6241	0.0004
42 Day Midline Shift	−0.5448	0.0033
1 Day Lesion Volume	−0.3433	0.0860
7 Day Lesion Volume	−0.3419	0.0749
42 Day Lesion Volume	−0.5770	0.0016
1 Day Swelling/Atrophy	−0.4084	0.0383
42 Day Swelling/Atrophy	0.1686	0.4005

## Data Availability

The authors declare that data associated with this paper are available upon request due to legal reasons.
